# Current role of nanoparticles in the treatment of lung cancer

**Published:** 2021-03-16

**Authors:** Eliseo Carrasco-Esteban, José Antonio Domínguez-Rullán, Patricia Barrionuevo-Castillo, Lira Pelari-Mici, Olwen Leaman, Sara Sastre-Gallego, Fernando López-Campos

**Affiliations:** ^1^Department Radiation Oncology, Hospital Central de la Defensa Gómez Ulla, Madrid, Spain; ^2^Department of Radiation Oncology, Hospital Universitario Ramón y Cajal, Madrid, Spain; ^3^Department of Radiation Oncology, Complejo Hospitalario de Jaén, Jaén, Spain; ^4^Department of Radiation Oncology, Hospital Universitario Gregorio Marañon, Madrid, Spain; ^5^Department of Radiation Oncology, Hospital Universitario Rey Juan Carlos, Madrid, Spain

**Keywords:** lung cancer, lipid nanoparticles, magnetic nanoparticles, polymer nanoparticles, drug delivery

## Abstract

**Background:**

Worldwide, lung cancer is one of the leading causes of cancer death. Nevertheless, new therapeutic agents have been developed to treat lung cancer that could change this mortality-rate. Interestingly, incredible advances have occurred in recent years in the development and application of nanotechnology in the detection, diagnosis, and treatment of lung cancer.

**Aim:**

Nanoparticles (NPs) have the ability to incorporate multiple drugs and targeting agents and therefore lead to an improved bioavailability, sustained delivery, solubility, and intestinal absorption.

**Relevance for patients:**

This review briefly summarizes the latest innovations in therapeutic nanomedicine in lung cancer with examples on magnetic, lipid, and polymer NP. Emphasis will be placed on future studies and ongoing clinical trials in this field.

## 1. Introduction

Lung cancer is the most common cancer in men and women in developed countries. It is also the leading cause of cancer death worldwide, causing 18.4% of all cancer deaths [1]. Approximately 70% of patients have advanced disease at the time of diagnosis, and only 15% of lung cancer patients are still alive 5 years after diagnosis [2]. There are several procedures for lung cancer diagnosis: Including physical exam, medical history, and imaging techniques such as X-ray, computed tomography (CT), bone scan, magnetic resonance imaging (MRI), positron emission tomography (PET), and combined PET-CT scan [3]. Among all imaging tools, combined PET-CT scan is the standard procedure for detecting the size and location of lung tumors, allowing accurate staging of disease, and determination of indeterminate lung nodules [4]. In addition to the early diagnosis of lung cancers, there is a need for an appropriate therapeutic strategy for the optimal treatment of these cancers. Several therapeutic procedures are commonly used for the treatment of lung cancer, including surgery, radiotherapy, radiosurgery, chemotherapy, and immunotherapy. The choice of the most appropriate treatment for lung cancer depends on the functional assessment of the patient, the stage, and the histological type of the disease. The gold standard for treating lung cancers is surgery, which is not suitable for metastatic and advanced stage lung cancers. When lung tumors cannot be resected due to spread to the surrounding tissues or when surgery is not necessary; traditionally, the best therapeutic option has been the combination of radiation and chemotherapy [5]. However, recent integration of targeted therapy and immunotherapy with these modalities has changed the treatment paradigm in these tumors [6].

Nanoparticles (NPs) are synthetic particles with a diameter of <100 nm that are generally derived from polymers, lipids, or metals such as gold. NPs have proven to be particularly useful in diverse medical applications, from diagnosis to cancer therapy [7]. The size of these NPs is remarkably similar to most of biological structures and molecules. Therefore, they confer functional properties for both *in vivo* and *in vitro* cancer research [8]. These NPs, if accompanied by biodegradable carriers, can be safely loaded with therapeutic compounds, to achieve concentrated local drug delivery with sustained release potential [9]. Due to these properties, they can enter the body cavities and the blood circulation for treatment with minimal invasion and improved bioavailability [10]. Furthermore, NPs have a larger surface/volume ratio than micro- and macro-sized particles, which enables them to be covered with several ligands at once leading to a higher drug loading and may facilitate interaction with different molecules, such as receptors present on the surface of target cells [11].

Throughout this review, we will discuss the potential use of NPs for the treatment of lung cancer. Some of these approaches have been evaluated in various clinical trials awaiting results or with recently published results, while most of them are still in clinical trials in the recruiting phase (Tables 1 and 2).

**Table 1:** Completed clinical trials with nanoparticles in lung cancer. An advanced search of ClinicalTrials.gov was performed in August 2020 for “nanoparticles and lung cancer.” These were reviewed and selected based on the status of the study.

Clinicaltrials.gov identifier (NCT number)	Study type	Description	Primary outcome	Planned enrollment (*n*)	Recruitment status
NCT01792479	Phase II	A phase II study to determine the safety and efficacy of BIND-014 (Docetaxel nanoparticles for injectable suspension) as second-line therapy to patients with NSCLC.	Objective response rate	64	Completed
NCT02283320	Phase II	BIND-014 (docetaxel nanoparticles for injectable suspension) is being studied in patients with v-Ki-ras2 Kirsten rat sarcoma viral oncogene homolog mutation positive or NSCLC who have progressed after treatment of one prior platinum-containing chemotherapy regimen.	Disease control rate	69	Completed
NCT00553462*	Phase II	This Phase II trial is studying how well giving carboplatin and paclitaxel albumin-stabilized nanoparticle formulation together with radiation therapy and erlotinib works in treating patients with Stage III NSCLC that cannot be removed by surgery.	Overall survival at 12 months	78	Completed
NCT00729612*	Phase II	This Phase II trial is studying how well paclitaxel albumin-stabilized nanoparticle formulation given together with carboplatin works in treating patients with Stage IIIB, Stage IV, or recurrent NSCLC.	Overall response rate	63	Completed
NCT00077246	Phase I-II	A Phase I/II trial studying the side effects and best dose of ABI-007 and to see how well it works in treating patients with stage IV NSCLC.	Maximum tolerated dose and dose-limiting toxicity of ABI-007 Objective target lesion response	64	Completed
NCT01380769*	Phase II	The purpose of this study is to compare median overall survival of patients with advanced NSCLC treated with CRLX101 to patients treated with best supportive care.	Overall survival	157	Completed
NCT02996214	Phase IV	Efficacy and the safety of paclitaxel liposome and cisplatin compared with gemcitabine and cisplatin as first-line therapy in advanced squamous NSCLC.	Progression free survival	536	Active, not recruiting
NCT02667743	Phase III	Efficacy and safety in first-line treatment in patients with advanced NSCLC with paclitaxel micelles for injection+cisplatin versus paclitaxel injection containing cremophor EL (polyoxyethylenated castor oil) + cisplatin.	Objective response rate	454	Active, not recruiting

* Published results, NSCLC: Non-small cell lung cancer

**Table 2:** Selected ongoing clinical trials with nanoparticles in lung cancer. An advanced search of ClinicalTrials.gov was performed in August 2020 for “nanoparticles and lung cancer.” These were reviewed and selected based on the status of the study.

Clinicaltrials.gov identifier (NCT number)	Study type	Description	Primary outcome	Planned enrollment (*n*)	Recruitment status
NCT04486833	Phase I-II	Safety and efficacy of GPX-001 + Osimertinib in NSCLC patients with activating EGFR mutations who have progressed while on treatment with osimertinib.	I- Maximum tolerated dose II- Progression-free Survival	100	Not yet recruiting
NCT04381910	Phase II	Efficacy and safety of LY01610 in patients with extensive-stage small cell lung cancer that progressed after first-line antitumor therapy.	Objective response rate and duration of response	90	Recruiting
NCT03088813	Phase II-III	Irinotecan liposome injection (ONIVYDE^®^) versus topotecan in patients with small cell lung cancer who have progressed on or after platinum-based First-Line therapy.	Overall survival	480	Recruiting
NCT04033354	Phase III	Clinical efficacy and safety of HLX10 + chemotherapy versus chemotherapy in subjects with locally advanced or metastatic squamous NSCLC who have not previously received systemic treatment.	Progression-free survival	516	Recruiting
NCT02769962	Phase I-II	Evaluate how safe it is to give CRLX101 and olaparib together and to see how well the combination treats a specific type of lung cancer called small cell lung cancer.	Objective response rate	123	Recruiting
NCT03670030	Phase II	Determine whether ABI-009 will make advanced, malignant neuroendocrine tumor (s) of the lung, gastrointestinal tract and/or pancreas that cannot be removed by surgery smaller and slow the spread of cancer in patients who have progressed or been intolerant to everolimus. All eligible participants will receive ABI-009.	Disease control rate	10	Recruiting

NSCLC: Non-small cell lung cancer

## 2. NPs types in lung cancer

### 2.1. Magnetic NPs (MNPs)

In the past two decades, MNPs have been studied for their potential use in the diagnosis and treatment of various diseases, and some formulations have been clinically approved for medical applications (e.g., LumirenVR, GastromarkVR, Feridex I.V.VR, and EndoremVR) [12,13]. Its high surface-to-volume ratio and the fact that they can be detected and manipulated by remote magnetic fields make them widely used in research. In concrete, according to a study recently published, NPs may allow an earlier and more accurate diagnosis in lung cancer [14]. A key advantage of the MNPs is the efficiency in the local deposition of drugs in the tumor and instead, has a minimum detrimental effect on healthy tissues [15].

The use of exogenous MNPs is limited by their potential toxicity, in part due to their biocompatibility. The biocompatibility of these particles depends on several factors: Structure and shape, size, concentration, surface properties, biodegradability, solubility, and pharmacokinetics, among others [16,17]. The size of NPs must be small enough (<200 nm) to extend the free circulation time in the blood and avoid being filtered by the spleen and liver, but at the same time, NPs must be larger than 10 nm to avoid rapid renal filtration [18]. In terms of surface properties, it has been established that MNPs with neutral surface charge exhibit longer circulation time and less uptake by the mononuclear phagocyte system due to decreased opsonization [19]. However, its tendency to agglomerate has forced the search for suitable surface coatings that allow MNPs to disperse into homogeneous ferrofluids, thus improving their stability [20]. The most commonly used biocompatible coating materials are polymers, liposomes, proteins, and inorganic materials [21,22]. In the absence of this biocompatibility, MNPs may disrupt cell metabolism and cause adverse effects. Several studies relate toxicity after exposure to these NPs to oxidative stress and reactive oxygen species generation [23,24]. Another form of cytotoxicity is caused by the increased concentration of free iron after the metabolization of iron oxide NPs [25]. The human body thoroughly regulates the total quantity of iron, so an excess concentration of this particle can be highly toxic. Therefore, we must avoid administration of these MNPs in high doses or in repeated doses in a short interval of time not to exceed the regulatory capacity of this material [26] and affect the cellular functionality of more sensitive organs such as heart, liver, or pancreatic beta cells [27]. Other forms of cytotoxicity reported are alterations of the cell cycle [28], alterations of the cytoskeleton [29], disruption of mitochondrial membrane potential [30], or decreased cell viability [31].

In this study, although we are focusing mainly in MNPS for the treatment of lung cancer, they also play a role in diagnosis. The immune superparamagnetic iron oxide NPs (SPIONs) are coated with oleic acid and carboxymethyl dextran, and then conjugated to mouse anti-CD44v6 monoclonal antibody, a protein marker for metastatic cancer. These NPs could be used as a T2 contrast agent to improve the detection of lung cancer metastasis on MRI [32]. Fluorescent particles can also be used as contrasts when conjugated with magnetic particles. Pfaff *et al*. reported the synthesis of galactose-displaying core-shell nanospheres by grafting a glycopolymer onto magnetic silica particles [33]. A method to functionalize the surface of SPIONs with a lung cancer-targeting peptide demonstrate high T2 relativity and the resulting NPs have shown specific targeting to the a_v_b_6_-positive H2009 cells, a peptide isolated from lung adenocarcinoma cells [34]. In addition, nebulized MNPs application can be useful in pulmonary imaging [35].

The use of heat for the treatment of diseases can be used in two ways: Thermoablation, when the temperature is raised high enough to cause immediate cellular death, and hyperthermia. Hyperthermia refers to mild temperature rises (40°C–45°C) that cause various forms of cellular damage and finally leads to apoptosis [36].

NP-based magnetically induced hyperthermia has been investigated in the treatment of glioblastoma multiforme [37], oral cancer [38], osteosarcoma [39], pancreatic cancer [40], breast cancer [41], melanoma [42], and prostate cancer [43]. In lung cancer, iron oxide MNPs were incorporated into magnetic nanocomposite microparticles through spray drying. These materials can be remotely heated in the presence of an alternating magnetic field and be used to trigger other therapies, increase transport of particles, and induce hyperthermia as a thermal therapy [44]. MNPs can also be attached to carriers like hydroxyapatite, a chemical structure with affinity for chemotherapy drugs and high biocompatibility. The iron and platinum incorporation to the hydroxyapatite creates a dual agent with chemo-hyperthermia properties with future application in the treatment of lung cancer [45]. The heating efficacy of polyacrylic acid coated MNP clusters under an alternating magnetic field was also studied, being able to radiosensitize NCI-H460 human lung cancer cells and effectively inhibited tumor growth *in vivo* [46]. MNPs may also achieve cell death in lung cancer cells in the presence of a pulsed electromagnetic field [47]. Another promising approach in the treatment of lung cancer is the use of ferucarbotran as inductive hyperthermia. Ferucarbotran is a MRI contrast agent reported to be able to generate heat in an alternating magnetic field [48]. The hyperthermia efficacy and cytotoxicity of porous MNPs coated by a polyethylene glycol (PEG) layer, and doxorubicin-loaded has been tested against human lung adenocarcinoma (A495) cells with promising results [49].

Another field of research is the use of systemic agents commonly used in lung cancer linked to MNPs to increase the response to these drugs. NPs, as drug delivery systems, allow the encapsulation of hydrophobic drugs, higher doses in the tumor microenvironment, less systemic distribution, and a reduction of side effects [50]. There are several clinically approved nanocarriers-based drugs, such as Doxil [51], Abraxane [52], and Onivyde [53]. An EGFR fragment and doxorubicin conjugated with MNPs increased the targeting ability and anti-cancer activity toward A549 cells (EGFR over-expressed cells present in human lung carcinoma cell line) [54]. Camptothecin shows cytotoxic effect against A549 cells line when conjugated with nickel ferrite NPs and cyclodextrins, which are cyclic sugar molecules with hydrophilic cover and hydrophobic cavity [55]. Short interfering RNA (siRNA) is another therapeutic agent used in cancer, which allows silencing genes, however, has a low penetration ability when is naked. SPIONs form complexes of siRNA with polypropyleneimine generation five dendrimers and PEG and target LHRH receptors, which are expressed in A549 human lung adenocarcinoma epithelial cell line [56]. Iron NPs encapsulated by carbon allows their loading with cisplatin, and these particles could be potentially used for concomitant therapy based on chemotherapy and hyperthermia [57]. Pemetrexed is a folate analog used in non-small cell lung cancer (NSCLC). Pemetrexed loaded magnetic O-Carboxymethyl chitosan NPs were synthesized for magnetic targeted treatment and the efficacy was examined by *in vivo* and *in vitro* studies. This NP has more cytotoxicity compared to free drug [58]. Alectinib is a second-generation anaplastic lymphoma kinase inhibitor, used in NSCLC. Alectinib has exceptionally low aqueous solubility, but a cascade dual-targeted polymeric nanocarrier (^DA^TAT-MNC_A,_ which contains Fe_3_O_4_) was capable of efficiently extravasation vessels into tumor tissue under the guidance of magnetic targeting [59].

Theranostic nanosystems are nanostructures that combine the diagnosis and therapeutic properties. Hybrid nanostructures that link Fe_3_O_4_ MNPs with polyelectrolyte layers and doxorubicin hydrochloride permit higher cytotoxicity toward A549 cells and can serve as T2-weighted MRI contrast [60]. Fe_3_O_4_ MNPs can also be coated with PEG that prevent recognition by mononuclear phagocyte system and extend the blood circulation duration [61]. Both molecular diagnosis and therapeutic treatment can be achieved with the use of polymeric micelles as nanocarriers. The hydrophobic core serves as depot for doxorubicin and SPIONs for therapeutic delivery and MRI applications, respectively [62]. At present, these studies have yielded promising results in the use of magnetic fields for diagnosis and treatment of lung cancer, although it has not been proven as an effective therapy in humans yet.

### 2.2. Polymer NPs

Polymeric NPs have been widely studied in cancer treatment [63]. Their chemical and physical properties make polymeric NPs attractive carriers for anticancer drugs. The development of NPs includes uncomplicated manipulation of particle size, surface charge, and the capability to encapsulate different targeting ligands into many functional groups, such as capsules, dendrimers, colloids, or micelles [64]. Both natural and synthetic polymers of different structures can be used. Most common natural polymers include polypeptides, albumin, gelatin, or chitosan and are frequently utilized because most of them are biocompatible and biodegradable. Synthetic polymers such as polyethylene PEG, poly lactic-co-glycolic acid (PLGA), poly lactic acid (PLA), and poly caprolactone are also employed [65]. NPs can be prepared by self-assembly of block copolymers with contrasting hydrophobicity between blocks. Most of them are suitable for different administration sites including intravenous, oral, nasal, or topic absorption [66].

Polymeric NPs have been used to address the major limitations of the drug delivery process; anti-neoplastic treatment side effects [67]. Their use of polymeric NP makes high concentration encapsulation of hydrophobic drugs possible, allowing the prolongation of circulation time and a more effective delivery at the target site, and demonstrating an improvement in the efficacy of chemotherapy and targeted agents [68]. Some evidence of relevant studies on the topic is summarized next. Abraxane^®^, an albumin-base nanocarrier loaded with paclitaxel has been approved by the FDA for the treatment of metastatic breast cancer and NSCLC [69]. PEG modified NPs carrying taxanes have demonstrated enhanced efficacy of combined chemoradiation in NSCLC *in vitro* and in an A549 human lung cancer cell line [70]. In 2017, Hu *et al*. reported on the efficacy of paclitaxel loaded poly caprolactone NPs in combination with chronomodulated chemotherapy and set out a potential role of circadian rhythms in tumor progression [71]. More recently, Wang *et al*. used mesenchymal stem cells as a carrier to improve drug delivery of NPs with paclitaxel and showed intercellular translocation of NPs to cancer cell with *in vivo* inhibited primary tumor growth [72]. The combination of gold nanorods with a polymer shell enhances its photothermal and thermoresponsive properties in a single nanocomposite with high biocompatibility and low cytotoxicity. The activation process shifts heat to near-infrared laser, thus, increasing the concentration of doxorubicin in the tumor and controlling the dose, time, and area under the curve [73].

The efficacy of nanocarriers for targeted therapy has also been explored. EGF peptides conjugated with gelatine NPs have shown greater cellular uptake than intravenous chemotherapy in lung adenocarcinoma cells in aerosol administration *in vivo* and *in vitro* [74]. Jiang *et al*. developed in 2015 a formulation of crizotinib (EML4-ALK fusion positive lung cancer) and polylactic tocopheryl PEG 1000 succinate (PLA-TPGS), which showed sustained release, induced cytotoxicity in NCIH3122 lung cancer cells and perceptible early and late apoptosis [75]. Erlotinib was loaded on PLGA as a strategy to overcome acquired resistance and has shown better loading efficiency, higher entrapment and sustained release [76]. In other study, afatinib and paclitaxel were loaded in PLGA inhaled microspheres. These NPs showed high biocompatibility and sustained lung concentration together with low concentration in other tissues. All these advantages make these targeted NP a good strategy for resistant lung-cancer [77].

Another type of NPs is lipid-based polymeric micelles, which are characterized by a structure composed of a hydrophobic core with the capacity to transport drugs and a hydrophilic PEG shell. T¡Bloodstream. life of these particles is longer than other NP¿ and as a result, accumulate in solid tumors after administration [78]. Genexol-PM is a micelle formulation of PLGA-b-methoxy PEG nanocarrier containing paclitaxel, approved for cancer therapy in South Korea and other European countries [79]. A Phase II trial of Genexol-PM in combination with gemcitabine in patients with advanced NSCLC demonstrated favorable anti-neoplastic results, but common Grade III-IV toxicities were observed [80]. Ccisplatin can be efficiently loaded to PEG polymeric NPs and showed enhanced anticancer efficacy against tumor cells *in vitro* [81]. Decreased toxicity has been seen with co-encapsulated micelles containing itraconazole and paclitaxel in the treatment of NSCLC [82]. Delivery of other agents like docetaxel in A549 NSCLC cell line is also possible using modified micelles covered by a-conotoxin [83]. Ding *et al*. developed a novel polyurethane micelle with both potential for MRI diagnosis and chemotherapy treatment [84]. The use of NPs in the delivery of chemotherapeutic agents has also been explored as a possible solution for drug resistance. Galactoxyloglucan and paclitaxel were used to synthetize biocompatible NPSs that were found to be highly effective in resistant A549 cells and could downregulate the expression of some multidrug-resistant proteins [85].

Polymeric NPs in the form of aerosol drugs have the potential to reduce systemic toxicity [86]. Lung administration of gelatin-based NPs has exhibited high anti-cancer activity of cisplatin in A549 lung adenocarcinoma cells [87]. Secondary cytotoxicity to lung cancer cells after 8 and 24 h has been shown when macrophages were treated with NPs (doxorubicin released from polyisobutyl cyanoacrylate) [88]. In another study, pulmonary delivery of hyaluronan-cisplatin conjugate showed increased lung drug concentration compared to iv cisplatin after 24 h, with lower tissue/plasma ratio in both kidneys and CNS, reducing dose-limiting toxicities [89].

On the other hand, the use of dendrimers has also been described in this setting. Dendrimers are synthetic polymers formed by repeated branched units emerging from a focal point and possess a large number of anionic, neutral, or cationic terminal functionalities exposed on the surface, resulting in hydrophilic or hydrophobic compounds [90]. They are nanometric molecules that are radially symmetric, globular, monodisperse, and homogeneous [91]. The properties of dendrimers are different to conventional polymers. The controllable and adjustable size, the interaction with cell membranes and various active drug molecules, and the characteristics of their internal structures and cavities, makes dendrimers excellent candidates for drug delivery systems [92,93]. The benefits of many drugs cannot be harnessed due to their poor solubility, toxicity, or stability problems. The use of dendrimers as carriers of these compositions can solve these problems, thus enhancing their clinical applications [94]. Chemotherapeutic drugs usually have a non-specific distribution, so that only a small part of the active agent reaches the site of action, and the pharmacokinetic characteristics are directly responsible for the *in situ* concentration of the drug and/or the active metabolite [95]. The transport capacity of dendrimers offers an advantage and represents an important strategy in cancer treatment, since dendrimers play the role of useful ligands for transporting the drug molecule to the tumor tissue throughout various biological compartments, while maximizing pharmacodynamic activity at the targeted site [96]. Poly(amidoamine) dendrimers (PAMAM) have been combined with various drugs indicated in lung cancer. Doxorubicin has been conjugated with fifth generation PAMAM dendrimers, with the advantage of increasing the therapeutic efficacy and specificity of action in lung cancer, directing the pH-controlled DOX-PEG-PAMAM dendrimer [97,98]. Imatinib conjugated with a PEGylated PAMAM G5 dendrimer has shown increased water solubility, and improved targeting and release in neoplastic cells [99,100]. Amreddy *et al*. developed and evaluated a NP system based on a folic acid (FA) conjugated PAMAM-dendrimer-polyethyleneimine system for the co-delivery of human receptor R, small interfering RNA (siRNA), and cisplatin for lung cancer therapy. They have proven that combination therapy using FA receptor (FAR) targeted dendrimer NPs exhibited specificity and selectivity toward FAR overexpressing cancer cells that improved the therapeutic efficiency and also reduced the cytotoxicity toward normal cells in an *in vitro* model of NSCLC and normal lung fibroblast [101].

### 2.3. Liposomes and solid lipid NPs

Liposomes are defined as spherical two-layer vesicular systems composed of phospholipids [102]. Studies regarding these vesicles are growing because of their ability to carry chemotherapy drugs, whether hydrophobic incorporated within the bimembrane or hydrophilic encapsulated within the aqueous core. Despite the multiple advantages of liposomes, such as their biocompatibility, absence of toxicity and biodegradation capacity, conventional liposomes tend to fuse together, which means less stability *in vivo* and faster degradation through the reticuloendothelial system (RES) [103].

To overcome these limitations, the surface of the liposomes has been modified by coating them with inert hydrophilic polymers, such as PEG. These polymers confer stability to the liposomal surface and give a protective layer that delays recognition by RES and therefore, increases their circulation time in the blood [104]. This modification has been used to encapsulate drugs such as doxorubicin. PEGylated liposomal doxorubicin has been validated in multiple Phase III studies for ovarian cancer with promising results [105].

Recently, solid lipid NPs, also known as submicron colloidal carriers, are on the rise due to their ability to transport both hydrophilic and lipophilic drugs, as well as their great stability, longer permanence in the bloodstream and possibility of being made up of biocompatible ingredients [106]. Over the years, interest in the use of liposomes in drug design has increased because of the benefits they provide, such as improved pharmacokinetic properties or reduced side effects of certain chemotherapeutic agents. In the management of NSCLC, platinum-based chemotherapy is the standard treatment in patients with locally advanced disease [107]. However, cisplatin is associated with acute nephrotoxicity in 20–40% of patients [108]. To reduce these side effects and improve response-rates Lipoplatin was developed. Cisplatin is encapsulated within a liposomal NP and has been shown to significantly reduce the occurrence of adverse events such as nephrotoxicity, peripheral neuropathy, ototoxicity, or myelopathy [109,110]. Stathopoulos *et al*. developed a randomized Phase III clinical trial to determine the efficacy of a combination treatment with Lipoplatin and paclitaxel versus cisplatin and paclitaxel in patients with advanced NSCLC, showing a statistically significant increase in the response rate in those patients treated with Lipoplatin [111].

Nonetheless, new controlled drug delivery systems at tumor sites are being investigated. One of them is lipid-polymer hybrid NPs, which combines the biocompatible properties of lipids and the structural advantages of polymers. Cisplatin-loaded lipid-chitosan hybrid NPs were formulated with promising results [112]. Subcellular drug targeting, particularly with platinum agents, has also been the subject of study in recent years. Paraskar *et al*. designed a lipid-based platinum complex which self-assembles into a NP with a pH-dependent cisplatin release, showing improvement in the antitumor efficacy of cisplatin [113]. Although both studies offered encouraging results, more pre-clinical studies are needed to demonstrate their efficacy.

Other types of chemotherapy agents commonly used are taxanes. One of the main problems of taxanes, such as paclitaxel, is their low solubility. Although historically this problem has been solved by administering the drug intravenously along with solubilizing agents such as Cremophor EL and Polysorbate 80, this usually leads to unwanted side effects such as hypersensitivity reactions, peripheral neuropathy, or myelosuppression [114]. With the aim of reducing these events and improve efficacy, liposomal paclitaxel was developed. A significant increase in the maximum tolerated dose has been observed in comparison with classical formulations [115]. If we focus on the treatment of lung cancer, there have been several studies using liposomal paclitaxel. In a Phase I clinical trial performed by Wang *et al.*, paclitaxel liposome was infused in NSCLC patients with malignant pleural effusions with promising results in terms of toxicity [116]. Given the potential of this formulation, clinical trials are underway to validate the efficacy and the safety of paclitaxel liposomes in conjunction with other drugs such as cisplatin as first-line therapy in advanced squamous NSCLC [117]. Attempts have also been made to improve efficacy and reduce side effects by using drugs such as doxorubicin or SN-38 encapsulated within micelles [118,119]. Studies are currently underway comparing paclitaxel micelles for injection in combination with cisplatin versus paclitaxel injection containing Cremophor EL as a first-line treatment of advanced NSCLC [120].

### 2.4. Metal NPs (MeNPs)

MeNPs are multi-purpose agents that offer numerous possibilities in different biomedical applications, such as diagnostic imaging [121], radiotherapy enhancement [122], and thermal ablation [123]. It is due to the strong electromagnetic field on the surface of the MeNP particles, wide optical properties, simplicity of the synthesis procedure, simple surface chemistry, and functionalization of the surface [124,125]. In addition, modification of the surface of MeNPs with biocompatible polymers (e.g., PEG) helps to increase the durability of drug action and is also used for targeted gene delivery and silencing purposes [126,127]. The physicochemical properties of MeNPs contribute to their potential for anticancer activity, which may be related to their intrinsic or extrinsic characteristics. Internal or intrinsic anti-tumor effects include their antioxidant activity, which decreases the rate of tumor progression [128,129].

Multiple MeNPs have been investigated in cancer therapy such as iron NPs [130,131], titanium dioxide NPs [132], zinc oxide NPs [133], cerium NPs [134], silver NPs [135], or gold NPs (AuNPs). AuNPs possess unique optical and surface properties, making them the main choice for researchers, especially in biological and pharmaceutical fields. AuNPs are colloidal or clustered particles consisting in an Au core and a surface coating. Size and shape control can be easily achieved to obtain AuNPs in the range of 1–150 nm with diverse morphologies that offer unique chemical, electrical, and optical properties. Among the available NPs, AuNPs have several advantages: They are biocompatible, can be synthesized in a wide range of sizes, and can be coated with a large number of molecules, including chemotherapy drugs [136-140].

Due to the optical properties of AuNPs, they are used especially in ultrasensitive detection and image-based therapeutic techniques required for the treatment of cancer. AuNPs have become promising vectors for cancer diagnosis and treatment [9,140,141]. Coelho *et al*. investigated the conjugation of bortezomib (BTZ), a proteasome inhibitor, conjugated with pegylated gold NPs (PEGAuNPs) in an *in vitro* model of pancreatic (S2-013), and lung (A549) cancer cell lines. They reported that conjugation with PEGAuNPs enhance the BTZ growth-inhibition effect on human cancer cells (S2-013 and A549) and decreases its toxicity on normal cells [142]. Ramalingam *et al*. have investigated the conjugation of doxorubicin (Dox) on the surface of AuNPs with polyvinylpyrrolidone (Dox@PVP-AuNPs) in lung cancer cells. They reported a significant cellular entry and intracellular release of Dox from the Dox@PVP-AuNPs complex with strong inhibition of lung cancer cell growth compared to free doxorubicin [143]. Afatinib (Afb), a chemotherapeutic drug approved for the treatment of EGFR positive lung cancer, has been conjugated with AuNPs, to improving drug efficacy and biocompatibility, administered to *in vitro* lung cancer cells. Afb-AuNPs were found to be up to 3.7 times more powerful when administered to lung cancer cells *in vitro* and were able to significantly inhibit cancer cell proliferation. In addition, when exposed to Afb-AuNP, human type I alveolar epithelial cells maintained viability and were found to release fewer pro-inflammatory cytokines compared to the free drug, demonstrating the biocompatibility of the conjugation [144].

NP-assisted radiation therapy is emerging as a promising modality of highly localized radiation boosting due to the photoelectric interaction of radiation therapy photons with high atomic (Z) number NPs, such as AuNPs [145]. This approach may allow for increased radiation dose delivery with minimal increase in toxicities to normal tissue [146]. However, delivering sufficiently high concentrations of such NPs to the tumor remains a challenge. Studies show that only up to 5% of NPs reach the lungs through the usual intravenous route [147]. Thus, many studies have concluded that boosting radiation from high-Z NPs would not be clinically significant for 6 MV radiotherapy, partly due to low concentrations of NPs that accumulate in the tumor when NPs are administered intravenously [148]. New approaches are being designed to bring higher concentrations of NPs to the tumor site. One of the lines of research focuses on the inhaled administration of NPs. Taratula *et al*. developed a special drug delivery system for delivery of NPs to lung tumors through inhalation route (IR). Their experimental results in animals showed that administering NPs by inhalation provides 3.5-14.6 times higher concentrations of NPs compared to the intravenous route. These studies included NPs of chemotherapy drugs such as cisplatin, which have a high-Z platinum component [149]. Hao *et al*. hypothesize that the administration of FDA approved concentrations of such platinum-based chemotherapy drugs through NP inhalation/instillation, which will allow for the delivery of concentrations powerful enough into the tumor to cause significant dose enhancement, through photoelectric mechanism during external beam radiotherapy (EBRT) with minimal toxicities to healthy tissue. Early results show major dose enhancement to lung tumors can be achieved using NPs with high-Z components administered through IR, in contrast to IV administration during EBRT [150].

### 2.5. Virus NPs

In the development of NPs, viruses (bacteriophages, plant, and mammalian viruses) have great potential thanks to their ability to transport materials within their capsid and efficient cell penetrability. Within the class of viruses, there are two main subgroups: Virus-like particles (VLPs) and viral NPs (VNPs) [62]. VLPs are considered non-infectious and are the genome-free versions of VNPs. The presence or absence of a viral genome can lead to different immunostimulatory profiles [151]. Plant virus-based NPs have been extensively used for targeted delivery of platinum-based chemotherapy, which is used in the treatment of a large percentage of cancer patients, including lung cancer [152].

Doxorubicin is an anthracycline used in several types of cancer, including small cell lung cancer. There are numerous studies that combine this antineoplastic with VNPs such as cucumber mosaic virus (FA-CMV-Dox), hepatitis B (Hb VLP) or potato virus X (PVX) to increase the drug delivery into the tumoral tissue [153-159]^.^ New drug delivery systems are recently being researched; one of these particles is the DOX-PhMV-PEG, which is obtained by loading the prodrug 6-maleimidocaproyl-hydrazone doxorubicin (DOX-EMCH) into the empty core of the physalis mottle virus (PhMV) and coating the external surface with PEG to improve biocompatibility. This particle showed *in vitro* stability and significantly higher efficacy *in vivo* compared to free doxorubicin in a mouse breast tumor model, laying the foundation for further investigations [160].

The role of VNPs in cancer diagnosis has also been investigated. Robertson *et al*. use the icosahedral head of the T4 bacteriophage linked to fluorescent dyes that allow the detection of A549 lung cancer cells line [161]. Tumor-specific targeting can be achieved with the use of specific ligands present in tumor cells. One of these is the FAR, which is found in greater quantity on the surface of tumor cells of the lungs, ovaries, kidneys, and others. The Cowpea mosaic virus can be attached to a FA-PEG conjugate, which allows for specific recognition of the cells that express the vitamin FA, allowing to target tumor cells *in vivo* [162,163].

Among the platforms that are being studied, the tobacco mosaic virus (TMV) offers the possibility of improving administration efficiency of drugs such as cisplatin, by loading the compound into the TMV cavity through a charge-driven reaction or by forming stable covalent adducts [164]. These complexes showed greater cytotoxicity compared to free cisplatin. In more recent studies, TMV coat protein was modified at specific sites with a molecular fluorous ponytail precipitating self-assembly into spherical NPs. These NPs were loaded with cisplatin through metal-ligand coordination, showing high stability [165]. Despite these promising results, it would be necessary to study the behavior of this TMV-cisplatin VNP complex in patients with lung cancer.

Researches are also oriented towards the use of viral nanotechnology as immunotherapy against cancer. The Cowpea mosaic virus interacts with the immune system and has the potential to act as an immunostimulatory agent by activating neutrophils in the tumor microenvironment. This property has been studied in lung cancer metastasis [166]. Trop-2 cell surface glycoprotein is overexpressed in lung cancer and has recently been used as an effective immunotherapeutic target along with the CD40 ligand (CD40L). In a recent investigation, researchers incorporated Trop-2 and CD40L into the membrane envelope of Human Immunodeficiency Virus (HIV) and VLPs for cancer vaccine development. Trop2CD40L VLPs generated specific humoral and cellular immune responses against Trop2, while reducing tumor growth in mice models. Due to its immunogenic properties and effects on the immune system, this particle represents a promising approach in lung cancer immunotherapy [167]. The overexpression of CD59, a membrane complement regulatory protein (mCRP), induces resistance to complement-dependent cytotoxicity (CDC) activation in lung cancer cells and consequently inhibits apoptosis. Therefore, investigators used VLPs of human JC polyomavirus (JCPyV) as a vector to carry a newly designed CD59-specific RNA expression plasmid driven by a lung-specific promoter (SP-B) for lung adenocarcinomas (pSPB-shCD59) to specifically inhibit CD59 overexpression in lung cancer cells. This study showed an 87% inhibition in tumor growth in mice that were injected with human lung cancer cells and a significant decrease in number and size of tumors in a mouse model of lung cancer metastasis. These results confirm the potential of this particle as a therapeutic agent for CD59 overexpressed lung cancer [168]. Virus-inspired polymer for endosomal release (VIPER) is a polymer system that imitates the mechanism of endosomal escape employed by adenovirus, this NP linked to siRNA, a molecule with the ability to knock down any gene involved in cancer development or progression, can be delivered to NCI-H1299 cells, a human NSCLC cell line [169].

The efficacy of VNPs as evidenced *in vivo* requires further understanding of the interactions with the immune system and biodistribution and structural studies, if it is to be translated into *in vivo* outcomes [170].

### 2.6. Quantum dots (QDs)

QDs are small nanocrystals, smallest of all NPs (3−30 nm) composed of semiconductor material made as an alloy nanocrystal colloid or core-shell from the metals from the periodic table. In general, QDs comprise the elements of Groups II-VI/III-V. Groups II-IV include elements such as cadmium–telluride, cadmium–selenide, zinc–selenide, and zinc sulfide, whereas Groups IIIV include the elements such as gallium arsenide, gallium nitride, indium arsenide, and indium phosphide [171-173]. Solubility and bioavailability of QDs are often a problem in drug discovery. The QDs can be adapted by coating with a polymer that improves solubility and absorbption [174]. The main problem with the clinical use of QDs is the potential to induce cytotoxicity [175]. This is explained by the fact that QDs are composed of metal atoms that are toxic and therefore can induce cytotoxicity by increasing the colloidal effect and the production of photon-induced free radicals [176]. QDs may play an essential role in the molecular profiling and cellular imaging of tumors to assist in the diagnosis and staging of disease, facilitating prognosis, and therapeutic use [174,177]. The unique optical properties of QDs, together with their capacity for functionalization with biomolecules, make QDs suitable candidates for the development of multimodal theranostic [178]. Ranjbar-Navazi *et al*. investigated the use of doxorrubicin conjugated with InP/ZnS QDs bi-functionalized with FA and D-glucosamine, in a cell culture of A549 human lung epithelial cancer cells and OVCAR-3 human ovarian cancer line. Their results showed that the conjugation of QDs with FA and GA can also enhance their uptake by both cells lines, while it can also decrease the cell cytotoxicity, and act as chemosensitizer against chemotherapy drugs including doxorubicin. In addition, they reported that multispectral analyses revealed a narrow fluorescent emission (570 nm) after excitation (400 nm). Hence, they have introduced a bi-functionalized doxorubicin-conjugated InP/ZnS QDs, which can be used as a theragnostic for simultaneous diagnosis and therapy of cancer [179]. Cai *et al*. reported a ZnO QDs-based pH-responsive drug delivery platform for intracellular controlled release of drugs. Doxorubicin (DOX) molecules were successfully loaded into PEG-functionalized ZnO QDs by formation of metal-DOX complex and covalent interactions. A targeting ligand, hyaluronic acid (HA), was conjugated with ZnO QDs to bind specifically to glycoprotein CD44 which is overexpressed by cancer cells. The pH-sensitive ZnO QDs were dissolved in Zn(2+) in the endosome/lysosome acid after being absorbed by the cancer cells, which triggered the release of the metal-drug complex and a controlled delivery of DOX. As a result, synergistic therapy was achieved due to the incorporation of the anti-tumor effect of Zn(2+) and DOX [180]. Finally, Erlotinib hydrochloride, a tyrosine kinase inhibitor, is a first-generation drug developed to treat NSCLC. Its active metabolite, desmethyl erlotinib (OSI-420), exhibits similar anticancer activity as erlotinib. Kulkarni *et al*. have conjugated OSI-420 to QDs and studied its activity on an *in vitro* model of A549 human lung cancer cells. They report a significantly better efficacy of conjugated QDs OSI-420 than pure drugs in all tested cell lines with similar cytotoxicity. Therefore, these results showed the conjugation of QDs and OSI-420 as an alternative to traditional anticancer therapy, by improving intracellular drug delivery [181].

## 3. Clinical Status of NPs in lung cancer

Advanced lung cancer tumors require in most cases combined chemotherapy and radiotherapy as standard of care, in addition to other emerging therapies such as immunotherapy or personalized medicine. However, combination paradigms and resistance patterns complicate the use of these agents. At this time, we have a large number of clinical trials under development, which try to incorporate the advances demonstrated in the field of NPs in these tumors to a clinical phase (Tables 1-2).

Some previous studies have reported the efficacy of nab-paclitaxel as a first-line chemotherapeutic agent for NSCLC [182-184]; however, to date, the efficacy of nab-paclitaxel in more advanced stages of the disease, or with other chemotherapy combinations has yet to be established. *(NCT00553462, NCT00729612)* Another interesting approach under study, after the results obtained in other tumor locations [185,186], is the use of BIND-014 (docetaxel NPs for injectable suspension) as a second line of treatment in metastatic lung cancer, still without published results *(NCT01792479, NCT02283320)*.

Preliminary promising results from the use of CRLX101 *NCT01380769*, a NP comprised of camptothecin conjugated to a cyclodextrin-based polymer, have been published [187]. CRLX101 is designed to increase the exposure of tumor cells to camptothecin while minimizing side effects. We will have to follow closely its development in the medium term, as well as the results of the studies that are currently in the recruitment period (table 1) with special attention to those in more advanced stages of development, *(NCT03088813, NCT04033354)* with potential to induce a change in the standard treatment.

## 4. Conclusion

NP-based medicine has infinite potential with novel applications being constantly developed for use in diagnosis, detection, imaging, and treatment of lung cancer. The development of different strategies for selective drug delivery to tumors and lung metastases depends on understanding the biology of the tumor, the microenvironment, and the interaction between malignant cells and NPs. There are several types of NPs that have been widely studied in the treatment of lung cancer. Some of them are now being actively investigated and are on the horizon facilitating personalized and tailored cancer treatment.

However, it is essential to investigate *in vivo* toxicity and biodistribution of NPs, as many of them are still in pre-clinical stages, being difficult to prepare NPs with different functionalities and homogenous size distribution to have better performance. When indicating the best nanoplatforms, it is necessary to carry out a selection with good reproducibility, simple preparation method, low-cost, and superior functional and structural properties for clinical applications, the safety and efficacy of NPs must still be proven in clinical trials. This review briefly summarizes the evidence in this field and can be a good tool to introduce researchers to the tremendous potential that this type of approach can bring to patients with lung cancer in the context of precision medicine in the medium term.

## References

[1] Bray F, Ferlay J, Soerjomataram I, Siegel RL, Torre LA, Jemal A (2018). Global Cancer Statistics 2018:GLOBOCAN Estimates of Incidence and Mortality Worldwide for 36 Cancers in 185 Countries. CA Cancer J Clin.

[2] Siegel RL, Miller KD, Jemal A (2018). Cancer Statistics, 2018. CA Cancer J Clin.

[3] Rivera MP, Mehta AC, Wahidi MM (2013). Establishing the Diagnosis of Lung Cancer:Diagnosis and Management of Lung Cancer:American College of Chest Physicians Evidence-based Clinical Practice Guidelines. Chest J.

[4] Hochhegger B, Alves GR, Irion KL, Fritscher CC, Fritscher LG, Concatto NH (2015). PET/CT Imaging in Lung Cancer:Indications and Findings. J Bras Pneumol.

[5] Kozower BD, Larner JM, Detterbeck FC, Jones DR (2013). Special Treatment Issues in Non-Small Cell Lung Cancer:Diagnosis and Management of Lung Cancer:American College of Chest Physicians Evidence-based Clinical Practice Guidelines. Chest.

[6] Ko EC, Raben D, Formenti SC (2018). The Integration of Radiotherapy with Immunotherapy for the Treatment of Non-Small Cell Lung Cancer. Clin Cancer Res.

[7] Horikoshi S, Serpone N, Horikoshi S, Serpone N (2013). Introduction to nanoparticles. Microwaves in Nanoparticle Synthesis:Fundamentals and Applications.

[8] Almeida JP, Lin AY, Langsner RJ, Eckels P, Foster AE, Drezek RA (2014). *In Vivo* Immune Cell Distribution of Gold Nanoparticles in Naive and Tumor Bearing Mice. Small.

[9] Chow EK, Ho D (2013). Cancer Nanomedicine:From Drug Delivery to Imaging. Sci Transl Med.

[10] Baetke SC, Lammers T, Kiessling F (2015). Applications of Nanoparticles for Diagnosis and Therapy of Cancer. Br J Radiol.

[11] Thanh NT, Green LA (2010). Functionalisation of Nanoparticles for Biomedical Applications. Nano Today.

[12] Avval ZM, Malekpour L, Raeisi F, Babapoor A, Mousavi SM, Hashemi SA (2020). Introduction of Magnetic and Supermagnetic Nanoparticles in New Approach of Targeting Drug Delivery and Cancer Therapy Application. Drug Metab Rev.

[13] Wu K, Su D, Liu J, Saha R, Wang JP (2019). Magnetic Nanoparticles in Nanomedicine:A Review of Recent Advances. Nanotechnology.

[14] Woodman C, Vundu G, George A, Wilson CM (2021). Applications and Strategies in Nanodiagnosis and nanotherapy in Lung Cancer. Semin Cancer Biol.

[15] Saadat M, Manshadi MK, Mohammadi M, Zare MJ, Zarei M, Kamali R (2020). Magnetic Particle Targeting for Diagnosis and Therapy of Lung Cancers. J Control Release.

[16] Singh N, Jenkins GJ, Asadi R, Doak SH (2010). Potential Toxicity of Superparamagnetic Iron Oxide Nanoparticles (SPION). Nano Rev.

[17] Ankamwar B, Lai TC, Huang JH, Liu RS, Hsiao M, Chen CH (2010). Biocompatibility of Fe_3_O_4_ Nanoparticles Evaluated Byin Vitrocytotoxicity Assays Using Normal, Glia and Breast Cancer Cells. Nanotechnology.

[18] Kandasamy G, Maity D (2015). Recent Advances in Superparamagnetic Iron Oxide Nanoparticles (SPIONs) for *In Vitro* and *In Vivo* Cancer Nanotheranostics. Int J Pharm.

[19] Aggarwal P, Hall JB, Mcleland CB, Dobrovolskaia MA, Mcneil SE (2009). Nanoparticle Interaction with Plasma Proteins as it Relates to Particle Biodistribution, Biocompatibility and Therapeutic Efficacy. Adv Drug Deliv Rev.

[20] Lu AH, Salabas EL, Schüth F (2007). Magnetic Nanoparticles:Synthesis, Protection, Functionalization, and Application. Angew Chem Int Ed Engl.

[21] Li X, Wei J, Aifantis KE, Fan Y, Feng Q, Cui FZ (2016). Current Investigations into Magnetic Nanoparticles for Biomedical Applications. J Biomed Mater Res Part A.

[22] Muthiah M, Park IK, Cho CS (2013). Surface Modification of Iron Oxide Nanoparticles by Biocompatible Polymers for Tissue Imaging and Targeting. Biotechnol Adv.

[23] Malvindi MA, De Matteis V, Galeone A, Brunetti V, Anyfantis GC, Athanassiou A (2014). Toxicity Assessment of Silica Coated Iron Oxide Nanoparticles and Biocompatibility Improvement by Surface Engineering. PLoS One.

[24] Dwivedi S, Siddiqui MA, Farshori NN, Ahamed M, Musarrat J, Al Khedhairy AA (2014). Synthesis, Characterization and Toxicological Evaluation of Iron Oxide Nanoparticles in Human Lung Alveolar Epithelial Cells. Colloids Surf B Biointerfaces.

[25] Soenen SJH, De Cuyper M (2009). Assessing Cytotoxicity of (Iron Oxide Based) Nanoparticles:An Overview of Different Methods Exemplified with Cationic Magnetoliposomes. Contrast Media Mol Imaging.

[26] Kunzmann A, Andersson B, Vogt C, Feliu N, Ye F, Gabrielsson S (2011). Efficient Internalization of Silica-Coated Iron Oxide Nanoparticles of Different Sizes by Primary Human Macrophages and Dendritic Cells. Toxicol Appl Pharmacol.

[27] Eaton JW, Qian M (2002). Molecular Bases of Cellular Iron Toxicity. Free Radic Biol Med.

[28] Wu J, Sun J (2011). Investigation on Mechanism of Growth Arrest Induced by Iron Oxide Nanoparticles in PC12 Cells. J Nanosci Nanotechnol.

[29] Wu W, Chen B, Cheng J, Wang J, Xu W, Liu L (2010). Biocompatibility of Fe_3_O_4_/DNR Magnetic Nanoparticles in the Treatment of Hematologic Malignancies. Int J Nanomed.

[30] Zhu MT, Wang Y, Feng WY, Wang B, Wang M, Ouyang H (2010). Oxidative Stress and Apoptosis Induced by Iron Oxide Nanoparticles in Cultured Human Umbilical Endothelial Cells. J Nanosci Nanotechnol.

[31] Wang D, Wang LH, Zhao Y, Lu YP, Zhu L (2010). Hypoxia Regulates the Ferrous Iron Uptake and Reactive Oxygen Species Level Via Divalent Metal Transporter 1 (DMT1) Exon1B by Hypoxia Inducible Factor-1. IUBMB Life.

[32] Wan X, Song Y, Song N, Li J, Yang L, Li Y (2016). The Preliminary Study of Immune Superparamagnetic Iron Oxide Nanoparticles for the Detection of Lung Cancer in Magnetic Resonance Imaging. Carbohydr Res.

[33] Pfaff A, Schallon A, Ruhland TM, Majewski AP, Schmalz H, Freitag R, Müller AH (2011). Magnetic and Fluorescent Glycopolymer Hybrid Nanoparticles for Intranuclear Optical Imaging. Biomacromolecules.

[34] Huang G, Zhang C, Li S, Khemtong C, Yang SG, Tian R (2009). A Novel Strategy for Surface Modification of Superparamagnetic Iron Oxide Nanoparticles for Lung Cancer Imaging. J Mater Chem.

[35] Nishimoto K, Mimura A, Aoki M, Banura N, Murase K (2015). Application of Magnetic Particle Imaging to Pulmonary Imaging Using Nebulized Magnetic Nanoparticles. Open J Med Imag.

[36] Chatterjee DK, Diagaradjane P, Krishnan S (2011). Nanoparticle-mediated Hyperthermia in Cancer Therapy. Ther Deliv.

[37] Gupta R, Sharma D (2019). Evolution of Magnetic Hyperthermia for Glioblastoma Multiforme Therapy. ACS Chem Neurosci.

[38] Legge CJ, Colley HE, Lawson MA, Rawlings AE (2019). Targeted Magnetic Nanoparticle Hyperthermia for the Treatment of Oral Cancer. J Oral Pathol Med.

[39] Liang B, Zuo D, Yu K, Cai X, Qiao B, Deng R (2020). Multifunctional Bone Cement for Synergistic Magnetic Hyperthermia Ablation and Chemotherapy of Osteosarcoma. Mater Sci Eng C Mater Biol Appl.

[40] Brero F, Albino M, Antoccia A, Arosio P, Avolio M, Berardinelli F (2020). Hadron Therapy, Magnetic Nanoparticles and Hyperthermia:A Promising Combined Tool for Pancreatic Cancer Treatment. Nanomaterials (Basel).

[41] Cędrowska E, Pruszyński M, Gawęda W, Żuk M, Krysiński P, Bruchertseifer F (2020). Trastuzumab Conjugated Superparamagnetic Iron Oxide Nanoparticles Labeled with 225Ac as a Perspective Tool for Combined a-Radioimmunotherapy and Magnetic Hyperthermia of HER2-Positive Breast Cancer. Molecules.

[42] Duval KE, Vernice NA, Wagner RJ, Fiering SN, Petryk JD, Lowry GJ (2019). Immunogenetic Effects of Low Dose (CEM43 30) Magnetic Nanoparticle Hyperthermia and Radiation in Melanoma Cells. Int J Hyperthermia.

[43] Attaluri A, Kandala SK, Wabler M, Zhou H, Cornejo C, Armour M (2015). Magnetic Nanoparticle Hyperthermia Enhances Radiation Therapy:A Study in Mouse Models of Human Prostate Cancer. Int J Hyperthermia.

[44] Stocke NA, Meenach SA, Arnold SM, Mansour HM, Hilt JZ (2015). Formulation and Characterization of Inhalable Magnetic Nanocomposite Microparticles (MnMs) for Targeted Pulmonary Delivery Via Spray Drying. Int J Pharm.

[45] Tseng CL, Chang KC, Yeh MC, Yang KC, Tang TP, Lin FH (2014). Development of a Dual-functional PtFeHAP Magnetic Nanoparticles Application for Chemo-hyperthermia Treatment of Cancer. Ceram Int.

[46] Ma J, Zhang Z, Zhang Z, Huang J, Qin Y, Li X (2015). Magnetic Nanoparticle Clusters Radiosensitise Human Nasopharyngeal and Lung Cancer Cells after Alternating Magnetic Field Treatment. Int J Hyperthermia.

[47] Baskar G, Ravi M, Panda JJ, Khatri A, Dev B, Santosham R (2017). Efficacy of Dipeptide-Coated Magnetic Nanoparticles in Lung Cancer Models Under Pulsed Electromagnetic Field. Cancer Invest.

[48] Araya T, Kasahara K, Nishikawa S, Kimura H, Sone T, Nagae H (2013). Antitumor Effects of Inductive Hyperthermia Using Magnetic Ferucarbotran Nanoparticles on Human Lung Cancer Xenografts in Nude Mice. Onco Targets Ther.

[49] Dabbagh A, Hedayatnasab Z, Karimian H, Sarraf M, Yeong CH, Hosseini HR (2019). Polyethylene Glycol-coated Porous Magnetic Nanoparticles for Targeted Delivery of Chemotherapeutics Under Magnetic Hyperthermia Condition. Int J Hyperthermia.

[50] Torchilin VP (2007). Targeted Pharmaceutical Nanocarriers for Cancer Therapy and Imaging. AAPS J.

[51] Barenholz Y (2012). Doxil®--the first FDA-approved nano-drug:Lessons Learned. J Control Release.

[52] Adrianzen Herrera D, Ashai N, Perez-Soler R, Cheng H (2019). Nanoparticle Albumin Bound-paclitaxel for Treatment of Advanced Non-small Cell Lung Cancer:An Evaluation of the Clinical Evidence. Expert Opin Pharmacother.

[53] Zhang H (2016). Onivyde for the Therapy of Multiple Solid Tumors. Onco Targets Ther.

[54] Kang MK, Mao W, Lee JB, Yoo HS (2017). Epidermal Growth Factor (EGF) Fragment-guided Anticancer Theranostic Particles for pH-responsive Release of Doxorubicin. Int J Pharm.

[55] Ramasamy S, Enoch IV, Rajkumar SR (2020). Polymeric Cyclodextrin-dextran Spooled Nickel Ferrite Nanoparticles:Expanded Anticancer Efficacy of Loaded Camptothecin. Mater Lett.

[56] Taratula O, Garbuzenko O, Savla R, Wang YA, He H, Minko T (2011). Multifunctional Nanomedicine Platform for Cancer Specific Delivery of siRNA by Superparamagnetic Iron Oxide Nanoparticles-Dendrimer Complexes. Curr Drug Deliv.

[57] Taylor A, Krupskaya Y, Krämer K, Füssel S, Klingeler R, Büchner B (2010). Cisplatin-loaded Carbon-encapsulated Iron Nanoparticles and their *In Vitro* Effects in Magnetic Fluid Hyperthermia. Carbon.

[58] Ak G, Aksu D, Çapkın E, Sarı Ö, Geboloğlu IK, Şanlıer ŞH (2020). Delivery of Pemetrexed by Magnetic Nanoparticles:Design, Characterization, *In Vitro* and *In Vivo* Assessment. Prep Biochem Biotechnol.

[59] Zeng F, Xu B, Zhu H, Wu S, Liao G, Xie D (2020). A Cascade Dual-targeted Nanocarrier for Enhanced Alectinib Delivery to ALK-Positive Lung Cancer. Biomater Sci.

[60] Zhao J, Li X, Wang X, Wang X (2019). Fabrication of Hybrid Nanostructures Based on Fe_3_O_4_ Nanoclusters as Theranostic Agents for Magnetic Resonance Imaging and Drug Delivery. Nanoscale Res Lett.

[61] Domac BH, AlKhatib S, Zirhli O, Akdogan NG, Dirican SC, Bulut G (2019). Effects of PEGylated Fe-Fe_3_O_4_ Core-Shell Nanoparticles on NIH3T3 and A549 Cell Lines. Heliyon.

[62] Guthi JS, Yang SG, Huang G, L Si, Khemtong C, Kessinger CW (2010). MRI-Visible Micellar Nanomedicine for Targeted Drug Delivery to Lung Cancer Cells. Mol Pharm.

[63] Hoffman A (2008). The Origins and Evolution of “Controlled”Drug Delivery Systems. J Control Release.

[64] Kong W, Sung D, Shim YH, Bae KH, Dubois P, Park TG (2009). Efficient Intracellular siRNA Delivery Strategy through Rapid and Simple Two Steps Mixing Involving Noncovalent Post-PEGylation. J Control Release.

[65] Chan JM, Valencia PM, Zhang L, Langer R, Farokhzad OC (2010). Polymeric Nanoparticles for Drug Delivery. Methods Mol Biol.

[66] Liu J, Liu J, Chu L, Wang Y, Duan Y, Feng L (2010). Novel Peptide-dendrimer Conjugates as Drug Carriers for Targeting Nonsmall Cell Lung Cancer. Int J Nanomedicine.

[67] Singh S, Nalwa HS (2007). Nanotechnology and Health Safety-toxicity and Risk Assessment of Nanostructured Material Son Human Health. J Nanosci Nanotechnol.

[68] Allouche J (2013). Synthesis of Organic and Bioorganic Nanoparticles:An Overview of the Preparation Methos. Nanomaterials:A Danger or a Promise.

[69] Ma P, Mumper RJ (2013). Paclitaxel Nano-delivery Systems:A Comprehensive Review. J Nanomed Nanotechnol.

[70] Jung J, Park SJ, Chung HK, Kang HW, Lee SW, Seo MH (2012). Polymeric Nanoparticles Containing Taxanes Enhance Chemoradiotherapeutic Efficacy in Non-small Cell Lung Cancer. Int J Radiat Oncol Biol Phys.

[71] Hu J, Fu S, Peng Q, Han YW, Xie J, Zan N (2017). Paclitaxel-Loaded Polymeric Nanoparticles Combined with Chronomodulated Chemotherapy on Lung Cancer:*In Vitro* and *In Vivo* Evaluation. Int J Pharm.

[72] Wang X, Chen H, Zeng X, Guo W, Jin Y, Wang S (2019). Efficient Lung Cancer-targeted Drug Delivery Via a Nanoparticle/MSC System. Acta Pharm Sin B.

[73] Zhang Z, Wang J, Nie X, Wen T, Ji Y, Wu X (2014). Near Infrared Laser-induced Targeted Cancer Therapy Using Thermoresponsive Polymer Encapsulated Gold Nanorods. J Am Chem Soc.

[74] Tseng CL, Su WY, Yen KC, Yang KC, Lin FH (2009). The Use of Biotinylated-EGF-Modified Gelatin Nanoparticle Carrier to Enhance Cisplatin Accumulation in Cancerous Lung Via Inhalation. Biomaterials.

[75] Jiang ZM, Dai SP, Xu YQ, Li T, Xie J, Li C (2015). Crizotinib-loaded Polymeric Nanoparticles in Lung Cancer Chemotherapy. Med Oncol.

[76] Vaidya B, Parvathaneni V, Kulkarni NS, Shukla SK, Damon JK, Sarode A (2019). Cyclodextrin Modified Erlotinib Loaded PLGA Nanoparticles for Improved Therapeutic Efficacy against Non-small Cell Lung Cancer. Int J Biol Macromol.

[77] Yang Y, Huang Z, Li J, Mo Z, Huang Y, Ma C (2019). PLGA Porous Microspheres Dry Powders for Codelivery of Afatinib-Loaded Solid Lipid Nanoparticles and Paclitaxel:Novel Therapy for EGFR Tyrosine Kinase Inhibitors Resistant Nonsmall Cell Lung Cancer. Adv Healthc Mater.

[78] Murakami M, Cabral H, Matsumoto Y, Wu S, Kano MR, Yamori T (2011). Improving Drug Potency and Efficacy by Nanocarrier-Mediated Subcellular Targeting. Sci Transl Med.

[79] Kim DW, Kim SY, Kim HK, Kim SW, Shin SW, Kim JS (2007). Multicenter Phase II Trial of Genexol-PM, a Novel Cremophor-free, Polymeric Micelle Formulation of Paclitaxel, with Cisplatin in Patients with Advanced Non-small-cell lung Cancer. Ann Oncol.

[80] Ahn HK, Jung M, Sym SJ, Shin DB, Kang SM, Kyung SY (2014). A phase II trial of Cremorphor EL-free paclitaxel (Genexol-PM) and gemcitabine in patients with advanced non-small cell lung cancer. Cancer Chemother Pharmacol.

[81] Li LP (2018). Cisplatin-loaded Polymeric Micelles with Aggregation-Induced Emission Feature for Cellular Imaging and Chemotherapy. Chem Eur.

[82] Zhang L, Liu Z, Kong C, Liu C, Yang K, Chen H (2018). Improving Drug Delivery of Micellar Paclitaxel against Non-Small Cell Lung Cancer by Co-Loading Itraconazole as a Micelle Stabilizer and a Tumor Vascular Manipulator. Small.

[83] Mei D, Zhao L, Chen B, Zhang X, Wang X, Yu Z (2018). a-Conotoxin Imi-modified Polymeric Micelles as Potential Nanocarriers for Targeted Docetaxel Delivery to a7-nAChR Overexpressed Non-small Cell Lung Cancer. Drug Deliv.

[84] Ding M, Zeng X, He X, Li J, Tan H, Fu Q (2014). Cell Internalizable and Intracellularly Degradable Cationic Polyurethane Micelles as a Potential Platform for Efficient Imaging and Drug Delivery. Biomacromolecules.

[85] Reshma P, Unnikrishnan B, Preethi GU, Syama HP, Archana MG, Remya K (2019). Overcoming Drug-resistance in Lung Cancer Cell by Paclitaxel Loaded Galactoxyloglucan Nanoparticles. Int J Biol Macromol.

[86] Abdelaziz H, Gaber M, Abd-Elwakil MM, Mabrouk MT, Elgohary MM, Kamel NM (2018). Inhalable Particulate Drug Delivery Systems for Lung Cancer Therapy:Nanoparticles, Microparticles, Nanocomposites and Nanoaggregates. J Control Release.

[87] Elzoghby AO (2013). Gelatin-based Nanoparticles as Drug and Gene Delivery Systems:Reviewing Three Decades of Research. J Control Release.

[88] Al-Hallak KM, Azarmi S, Anwar-Mohamed A, Roa WH, Löbenberg R (2010). Secondary Cytotoxicity Mediated by Alveolar Macrophages:A Contribution to the Total Efficacy of Nanoparticles in Lung Cancer Therapy?. Eur J Pharm Biopharm.

[89] Xie Y, Aillon KL, Cai S, Christian JM, Davies NM, Berkland CJ (2010). Pulmonary Delivery of Cisplatin-hyaluronan Conjugates Via Endotracheal Instillation for the Treatment of Lung Cancer. Int J Pharm.

[90] Lyu Z, Ding L, Huang AY, Kao CL, Peng L (2019). Poly(amidoamine)dendrimers:Covalent and supramolecular synthesis. Mater Today Chem.

[91] Sohail I, Bhatti IA, Ashar A, Sarim FM, Mohsin M, Naveed R (2020). Polyamidoamine (PAMAM) Dendrimers Synthesis, Characterization and Adsorptive Removal of Nickel Ions from Aqueous Solution. J Mater Res Technol.

[92] Pandita D, Poonia N, Kumar S, Lather V, Madaan K (2014). Dendrimers in Drug Delivery and Targeting:Drug-dendrimer Interactions and Toxicity Issues. J Pharm Bioallied Sci.

[93] Cojocaru FD, Botezat D, Gardikiotis I, Uritu CM, Dodi G, Trandafir L (2020). Nanomaterials Designed for Antiviral Drug Delivery Transport Across Biological Barriers. Pharmaceutics.

[94] Sherje AP, Jadhav M, Dravyakar BR, Kadam D (2018). Dendrimers:A Versatile Nanocarrier for Drug Delivery and Targeting. Int J Pharm.

[95] Gerber DE (2008). Targeted Therapies:A New Generation of Cancer Treatments. Am Fam Physician.

[96] Castro RI, Forero-Doria O, Guzmán L (2018). Perspectives of Dendrimer-based Nanoparticles in Cancer Therapy. An Acad Bras Cienc.

[97] Zhong Q, Da Rocha SR (2016). Poly(Amidoamine) Dendrimer-doxorubicin Conjugates:*In Vitro* Characteristics and Pseudosolution Formulation in Pressurized Metered-Dose Inhalers. Mol Pharm.

[98] Kale AA, Torchilin VP (2007). Design, Synthesis, and Characterization of pH-Sensitive PEG-PE Conjugates for Stimuli-sensitive Pharmaceutical Nanocarriers:The Effect of Substitutes at the Hydrazone Linkage on the pH Stability of PEG-PE Conjugates. Bioconjug Chem.

[99] Markowicz M, Szymánski P, Ciszewski M, Klys A, Mikiciuk-Olasik E (2012). Evaluation of Poly(Amidoamine) Dendrimers as Potential Carriers of Iminodiacetic Derivatives Using Solubility Studies and 2D-NOESY NMR Spectroscopy. J Biol Phys.

[100] Karthikeyan R, Vijayarajkumar P (2015). PEGylated Nanoarchitechture Mediated Solubility Enhancement of Tyrosine-kinase Inhibitor. Inventi Rapid.

[101] Amreddy N, Babu A, Panneerselvam J, Srivastava A, Muralidharan R, Chen A (2018). Chemo-biologic Combinatorial Drug Delivery Using Folate Receptor-targeted Dendrimer Nanoparticles for Lung Cancer Treatment. Nanomedicine.

[102] Abu Lila AS, Ishida T (2017). Liposomal Delivery Systems:Design Optimization and Current Applications. Biol Pharm Bul Biol Pharm Bull.

[103] Haluska CK, Riske KA, Marchi-Artzner V, Lehn JM, Lipowsky R, Dimova R (2006). Time Scales of Membrane Fusion Revealed by Direct Imaging of Vesicle Fusion with High Temporal Resolution. Proc Natl Acad Sci.

[104] Torchilin VP (2005). Recent Advances with Liposomes as Pharmaceutical Carriers. Nat Rev Drug Discov.

[105] Wagner U, Marth C, Largillier R, Kaern J, Brown C, Heywood M (2012). Final Overall Survival Results of Phase III GCIG CALYPSO Trial of Pegylated Liposomal Doxorubicin and Carboplatin vs Paclitaxel and Carboplatin in Platinum-sensitive Ovarian Cancer Patients. Br J Cancer.

[106] Paliwal R, Paliwal SR, Kenwat R, Kurmi BD, Sahu MK (2020). Solid Lipid Nanoparticles:A Review on Recent Perspectives and Patents. Expert Opin Ther Pat.

[107] Aupérin A, Péchoux CL, Rolland E, Curran WJ, Furuse K, Fournel P (2010). Meta-Analysis of Concomitant Versus Sequential Radiochemotherapy in Locally Advanced Non-Small-Cell Lung Cancer. J Clin Oncol.

[108] Hamroun A, Lenain R, Bigna JJ, Speyer E, Bui L, Chamley P (2019). Prevention of Cisplatin-induced Acute Kidney Injury:A Systematic Review and Meta-analysis. Drugs.

[109] Boulikas T (2009). Clinical Overview on Lipoplatin™:A Successful Liposomal Formulation of Cisplatin. Expert Opin Investig Drugs.

[110] Giuberti CD, Reis EC, Rocha TG, Leite EA, Lacerda RG, Ramaldes GA (2010). Study of the Pilot Production Process of Long-Circulating and pH-sensitive Liposomes Containing Cisplatin. J Liposome Res.

[111] Stathopoulos GP, Antoniou D, Dimitroulis J, Stathopoulos J, Marosis K, Michalopoulou P (2011). Comparison of Liposomal Cisplatin Versus Cisplatin in Non-squamous Cell Non-small-cell Lung Cancer. Cancer Chemother Pharmacol.

[112] Khan MM, Madni A, Torchilin V, Filipczak N, Pan J, Tahir N (2019). Lipid-Chitosan Hybrid Nanoparticles for Controlled Delivery of Cisplatin. Drug Deliv.

[113] Paraskar AS, Soni S, Chin KT, Chaudhuri P, Muto KW, Berkowitz J (2010). Harnessing Structure-activity Relationship to Engineer a Cisplatin Nanoparticle for Enhanced Antitumor Efficacy. Proc Natl Acad Sci.

[114] Khurana RK, Mahajan M, Teenu Kapoor S, Jain S, Singh B (2016). The Sojourn from Parenteral to Oral Taxanes using Nanocarrier Systems:A Patent Review. Recent Pat Drug Deliv Formul.

[115] Koudelka Š, Turánek J (2012). Liposomal Paclitaxel Formulations. J Controlled Release.

[116] Wang X, Zhou J, Wang Y, Zhu Z, Lu Y, Wei Y (2010). A Phase I Clinical and Pharmacokinetic Study of Paclitaxel Liposome Infused in Non-small Cell Lung Cancer Patients with Malignant Pleural Effusions. Eur J Cancer.

[117] Paclitaxel Liposome for Squamous Non-Small-cell Lung Cancer Study (LIPUSU) Full Text View Paclitaxel Liposome for Squamous Non-Small-cell Lung Cancer Study (LIPUSU) Full Text View.

[118] Matsumura Y, Hamaguchi T, Ura T, Muro K, Yamada Y, Shimada Y (2004). Phase I Clinical Trial and Pharmacokinetic Evaluation of NK911, a Micelle-Encapsulated Doxorubicin. Br J Cancer.

[119] Hamaguchi T, Doi T, Eguchi-Nakajima T, Kato K, Yamada Y, Shimada Y (2010). Phase I Study of NK012, a Novel SN-38-Incorporating Micellar Nanoparticle, in Adult Patients with Solid Tumors. Clin Cancer Res.

[120] Paclitaxel Micelles for Injection / Paclitaxel Injection in Combination with Cisplatin for First-line Therapy of Advanced NSCLC Full Text View (2020). Full Text View ClinicalTrials.

[121] Selvan ST, Tan TT, Yi DK, Jana NR (2010). Functional and Multifunctional Nanoparticles for Bioimaging and Biosensing. Langmuir.

[122] Hainfeld JF, Dilmanian FA, Slatkin DN, Smilowitz HM (2008). Radiotherapy Enhancement with Gold Nanoparticles. J Pharm Pharmacol.

[123] Yavuz MS, Cheng Y, Chen J, Cobley CM, Zhang Q, Rycenga M (2009). Gold Nanocages Covered by Smart Polymers for Controlled Release with Near-infrared Light. Nat Mater.

[124] Xie J, Lee S, Chen X (2010). Nanoparticle-based Theranostic Agents. Adv Drug Deliv Rev.

[125] Sperling RA, Parak W (2010). Surface Modification, Functionalization and Bioconjugation of Colloidal Inorganic Nanoparticles. Philos Trans R Soc Lond A Math Phys Eng Sci.

[126] Sau TK, Rogach AL, Jäckel F, Klar TA, Feldmann J (2010). Properties and Applications of Colloidal Nonspherical Noble Metal Nanoparticles. Adv Mater.

[127] Sperling RA, Gil PR, Zhang F, Zanella M, Parak WJ (2008). Biological Applications of Gold Nanoparticles. Chem Soc Rev.

[128] Porcel E, Liehn S, Remita H, Usami N, Kobayashi K, Furusawa Y (2010). Platinum Nanoparticles:A Promising Material for Future Cancer Therapy?. Nanotechnology.

[129] Danhier F, Feron O, Préat V (2010). To Exploit the Tumor Microenvironment:Passive and Active Tumor Targeting of Nanocarriers for Anti-cancer Drug Delivery. J Control Release.

[130] Silva AC, Oliveira TR, Mamani JB, Malheiros SM, Malavolta L, Pavon LF (2011). Application of Hyperthermia Induced by Superparamagnetic Iron Oxide Nanoparticles in Glioma Treatment. Int J Nanomed.

[131] Maier-Hauff K, Ulrich F, Nestler D, Niehoff H, Wust P, Thiesen B (2011). Efficacy and Safety of Intratumoral Thermotherapy Using Magnetic Iron-oxide Nanoparticles Combined with External Beam Radiotherapy on Patients with Recurrent Glioblastoma Multiforme. J Neurooncol.

[132] Hou Z, Zhang Y, Deng K, Chen Y, Li X, Deng X (2015). UV-emitting Upconversionbased TiO_2_ Photosensitizing Nanoplatform:Near-infrared Light Mediated *In Vivo* Photodynamic Therapy Via Mitochondria Involved Apoptosis Pathway. ACS Nano.

[133] Baskar G, Chandhuru J, Fahad KS, Praveen AS, Chamundeeswari M, Muthukumar T (2015). Anticancer Activity of Fungal l-Asparaginase Conjugated with Zinc Oxide Nanoparticles. J Mater Sci Mater Med.

[134] Ali D, Alarifi S, Alkahtani S, AlKahtane AA, Almalik A (2015). Cerium Oxide Nanoparticles Induce Oxidative Stress and Genotoxicity in Human Skin Melanoma Cells. Cell Biochem Biophys.

[135] Zhang XF, Liu ZG, Shen W, Gurunathan S (2016). Silver Nanoparticles:Synthesis, Characterization, Properties, Applications, and Therapeutic Approaches. Int J Mol Sci.

[136] Haume K, Rosa S, Grellet S, ´Smiałek MA, Butterworth KT, Solov'yov AV (2016). Gold Nanoparticles for Cancer Radiotherapy:A Review. Cancer Nano.

[137] Lopez-Campos F, Candini D, Carrasco E, Francés MA (2019). Nanoparticles Applied to Cancer Immunoregulation. Rep Pract Oncol Radiother.

[138] Zhao N, Pan Y, Cheng Z, Liu H (2016). Gold Nanoparticles for Cancer Theranostics a Brief Update. J Innov Opt Health Sci.

[139] Zhao P, Li N, Astruc D (2013). State of the Art in Gold Nanoparticle Synthesis. Coord Chem Rev.

[140] Singh P, Pandit S, Mokkapati VR, Garg A, Ravikumar V, Mijakovic I (2018). Gold Nanoparticles in Diagnostics and Therapeutics for Human Cancer. Int J Mol Sci.

[141] Mioc A, Mioc M, Ghiulai R, Voicu M, Racoviceanu R, Trandafirescu C (2019). Gold Nanoparticles as Targeted Delivery Systems and Theranostic Agents in Cancer Therapy. Curr Med Chem.

[142] Coelho SC, Almeida GM, Santos-Silva F, Pereira MC, Coelho MA (2016). Enhancing the Efficiency of Bortezomib Conjugated to Pegylated Gold Nanoparticles:An *In Vitro* Study on Human Pancreatic Cancer Cells and Adenocarcinoma Human Lung Alveolar Basal Epithelial Cells. Expert Opin Drug Deliv.

[143] Ramalingam V, Varunkumar K, Ravikumar V, Rajaram R (2018). Target Delivery of Doxorubicin Tethered with PVP Stabilized Gold Nanoparticles for Effective Treatment of Lung Cancer. Sci Rep.

[144] Cryer AM, Chan C, Eftychidou A, Maksoudian C, Mahesh M, Tetley TD (2019). Tyrosine Kinase Inhibitor Gold Nanoconjugates for the Treatment of Non-Small Cell Lung Cancer. ACS Appl Mater Interfaces.

[145] Gadoue SM, Toomeh D (2019). Radio-sensitization Efficacy of Gold Nanoparticles in Inhalational Nanomedicine and the Adverse Effect of Nano-Detachment due to Coating Inactivation. Phys Med.

[146] Ngwa W, Kumar R, Sridhar S, Korideck H, Zygmanski P, Cormack RA (2014). Targeted Radiotherapy with Gold Nanoparticles:Current Status and Future Perspectives. Nanomedicine (Lond).

[147] Taratula O, Garbuzenko OB, Chen AM, Minko T (2011). Innovative Strategy for Treatment of Lung Cancer:Targeted Nanotechnology-based Inhalation Co-delivery of Anticancer Drugs and siRNA. J Drug Target.

[148] Rousseau J, Barth RF, Fernandez M, Adam JF, Balosso J, Esteve F (2010). Efficacy of Intracerebral Delivery of Cisplatin in Combination with Photon Irradiation for Treatment of Brain Tumors. J Neurooncol.

[149] Taratula O, Kuzmov A, Shah M, Garbuzenko OB, Minko T (2013). Nanostructured Lipid Carriers as Multifunctional Nanomedicine Platform for Pulmonary Co-delivery of Anticancer Drugs and siRNA. J Control Release.

[150] Hao Y, Altundal Y, Moreau M, Sajo E, Kumar R, Ngwa W (2015). Potential for Enhancing External Beam Radiotherapy for Lung Cancer Using High-Z Nanoparticles Administered Via Inhalation. Phys Med Biol.

[151] Wang C, Beiss V, Steinmetz NF (2019). Cowpea Mosaic Virus Nanoparticles and Empty Virus-Like Particles Show Distinct but Overlapping Immunostimulatory Properties. J Virol.

[152] Johnstone TC, Suntharalingam K, Lippard SJ (2016). The Next Generation of Platinum Drugs:Targeted Pt(II) Agents, Nanoparticle Delivery, and Pt(IV) Prodrugs. Chem Rev.

[153] Chung YH, Cai H, Steinmetz NF (2020). Viral Nanoparticles for Drug Delivery, Imaging, Immunotherapy, and Theranostic Applications. Adv Drug Deliv Rev.

[154] Ren Y, Wong SM, Lim LY (2007). Folic Acid-conjugated Protein Cages of a Plant Virus:A Novel Delivery Platform for Doxorubicin. Bioconjug Chem.

[155] Zeng Q, Wen H, Wen Q, Chen X, Wang Y, Xuan W (2013). Cucumber Mosaic Virus as Drug Delivery Vehicle for Doxorubicin. Biomaterials.

[156] Aljabali AA, Shukla S, Lomonossoff GP, Steinmetz NF, Evans DJ (2013). CPMV-DOX Delivers. Mol Pharm.

[157] Cao J, Guenther RH, Sit TL, Opperman CH, Lommel SA, Willoughby JA (2014). Loading and Release Mechanism of Red Clover Necrotic Mosaic Virus Derived Plant Viral Nanoparticles for Drug Delivery of Doxorubicin. Small.

[158] Le DH, Lee KL, Shukla S, Commandeur U, Steinmetz NF (2017). Potato Virus X, a Filamentous Plant Viral Nanoparticle for Doxorubicin Delivery in Cancer Therapy. Nanoscale.

[159] Shan W, Zhang D, Wu Y, Lv X, Hu B, Zhou X (2018). Modularized Peptides Modified HBc Virus-like Particles for Encapsulation and Tumor-targeted Delivery of Doxorubicin. Nanomedicine.

[160] Hu H, Steinmetz NF (2020). Doxorubicin-Loaded Physalis Mottle Virus Particles as a pH-Responsive Prodrug for Cancer Therapy. Biotechnol J.

[161] Robertson KL, Soto CM, Archer MJ, Odoemene O, Liu JL (2011). Engineered T4 Viral Nanoparticles for Cellular Imaging and Flow Cytometry. Bioconjug Chem.

[162] Destito G, Yeh R, Rae CS, Finn MG, Manchester M (2007). Folic Acid-mediated Targeting of Cowpea Mosaic Virus Particles to Tumor Cells. Chem Biol.

[163] Beatty PH, Lewis JD (2019). Cowpea Mosaic Virus Nanoparticles for Cancer Imaging and Therapy. Adv Drug Deliv Rev.

[164] Franke CE, Czapar AE, Patel RB, Steinmetz NF (2018). Tobacco Mosaic Virus-Delivered Cisplatin Restores Efficacy in Platinum-Resistant Ovarian Cancer Cells. Mol Pharm.

[165] Gao S, Liu X, Wang Z, Jiang S, Wu M, Tian Y (2018). Fluorous Interaction Induced Self-assembly of Tobacco Mosaic Virus Coat Protein for Cisplatin Delivery. Nanoscale.

[166] Lizotte PH, Wen AM, Sheen MR, Fields J, Rojanasopondist P, Steinmetz NF (2016). *In Situ* Vaccination with Cowpea Mosaic Virus Nanoparticles Suppresses Metastatic Cancer. Nat Nanotechnol.

[167] Xi W, Ke D, Min L, Lin W, Jiahui Z, Fang L (2018). Incorporation of CD40 Ligand Enhances the Immunogenicity of Tumor-Associated Calcium Signal Transducer 2 Virus-Like Particles Against Lung Cancer. Int J Mol Med.

[168] Lin MC, Shen CH, Chang D, Wang M (2019). Inhibition of Human Lung Adenocarcinoma Growth and Metastasis by JC Polyomavirus-like Particles Packaged with an SP-B Promoter-Driven CD59-Specific shRNA. Clin Sci.

[169] Feldmann DP, Cheng Y, Kandil R, Xie Y, Mohammadi M, Harz H (2018). *In Vitro* and *In Vivo* Delivery of siRNA via VIPER Polymer System to Lung Cells. J Control Release.

[170] Sainsbury F (2017). Virus-like Nanoparticles:Emerging Tools for Targeted Cancer Diagnostics and Therapeutics. Ther Deliv.

[171] Lewinski N, Colvin V, Drezek R (2008). Cytotoxicity of Nanoparticles. Small.

[172] de Mello DonegáC (2011). Synthesis and Properties of Colloidal Heteronanocrystals. Chem Soc Rev.

[173] Ghasemi Y, Peymani P, Afifi S (2009). Quantum Dot:Magic Nanoparticle for Imaging, Detection and Targeting. Acta Biomed.

[174] Jin S, Hu YX, Gu ZJ, Liu L, Wu HC (2011). Application of Quantum Dots in Biological Imaging. J Nanomater.

[175] Hardman R (2006). A Toxicologic Review of Quantum Dots:Toxicity Depends on Physicochemical and Environmental Factors. Environ Health Perspect.

[176] Smith AM, Nie S (2009). Next-generation Quantum Dots. Nat Biotechnol.

[177] Singh RD, Shandilya R, Bhargava A, Kumar R, Tiwari R, Chaudhury K (2018). Quantum Dot Based Nano-Biosensors for Detection of Circulating Cell Free miRNAs in Lung Carcinogenesis:From Biology to Clinical Translation. Front Genet.

[178] Mashinchian O, Johari-Ahar M, Ghaemi B, Rashidi M, Barar J, Omidi Y (2014). Impacts of Quantum Dots in Molecular Detection and Bioimaging of Cancer. Bioimpacts.

[179] Ranjbar-Navazi Z, Eskandani M, Johari-Ahar M, Nemati A, Akbari H, Davaran S (2018). Doxorubicin-conjugated D-glucosamine-and Folate- bi-functionalised InP/ZnS Quantum Dots for Cancer Cells Imaging and Therapy. J Drug Target.

[180] Cai X, Luo Y, Zhang W, Du D, Lin Y (2016). pH-Sensitive ZnO Quantum Dots-Doxorubicin Nanoparticles for Lung Cancer Targeted Drug Delivery. ACS Appl Mater Interfaces.

[181] Kulkarni NS, Parvathaneni V, Shukla SK, Barasa L, Perron JC, Yoganathan S (2019). Tyrosine Kinase Inhibitor Conjugated Quantum Dots for Non-small Cell Lung Cancer (NSCLC) Treatment. Eur J Pharm Sci.

[182] Socinski MA, Bondarenko I, Karaseva NA, Makhson AM, Vynnychenko I, Okamoto I (2012). Weekly Nab-paclitaxel in Combination with Carboplatin Versus Solvent-based Paclitaxel Plus Carboplatin as First-line Therapy in Patients with Advanced Non-small Cell Lung Cancer:Final Results of a Phase III Trial. J Clin Oncol.

[183] Yang JJ, Huang C, Chen GY, Song Y, Cheng Y, Yan HH (2014). A Randomized Phase II Clinical Trial of Nab-paclitaxel and Carboplatin Compared with Gemcitabine and Carboplatin as First-line Therapy in Locally Advanced or Metastatic Squamous Cell Carcinoma of Lung. BMC Cancer.

[184] Langer CJ, Hirsh V, Ko A, Renschler MF, Socinski MA (2015). Weekly Nab-paclitaxel in Combination with Carboplatin as First-line Therapy in Patients with Advanced Non-small Cell Lung Cancer:Analysis of Safety and Efficacy in Patients with Renal Impairment. Clin Lung Cancer.

[185] Autio KA, Dreicer R, Anderson J, Garcia JA, Alva A, Hart LL (2018). Safety and Efficacy of BIND-014, a Docetaxel Nanoparticle Targeting Prostate-Specific Membrane Antigen for Patients With Metastatic Castration-Resistant Prostate Cancer:A Phase 2 Clinical Trial. JAMA Oncol.

[186] Von Hoff DD, Mita MM, Ramanathan RK, Weiss GJ, Mita AC, LoRusso PM (2016). Phase I Study of PSMA-Targeted Docetaxel-Containing Nanoparticle BIND-014 in Patients with Advanced Solid Tumors. Clin Cancer Res.

[187] Chen YF, Wang YH, Lei CS, Changou CA, Davis ME, Yen Y (2019). Host Immune Response to Anti-cancer Camptothecin Conjugated Cyclodextrin-based Polymers. J Biomed Sci.

